# Validation of a novel surveillance paradigm for ventilator-associated events

**DOI:** 10.1186/cc12902

**Published:** 2013-11-05

**Authors:** Peter MC Klein Klouwenberg, Maaike SM van Mourik, David SY Ong, Janneke Horn, Marcus J Schultz, Olaf L Cremer, Marc JM Bonten

**Affiliations:** 1 Department of Medical Microbiology, University Medical Center Utrecht, the Netherlands; 2 Department of Intensive Care, University Medical Center Utrecht, the Netherlands; 3 Department of Intensive Care Medicine, Academic Medical Center, University of Amsterdam, the Netherlands; 4 Julius Center for Health Sciences and Primary Care, University Medical Center Utrecht, the Netherlands

## Background

Reliable surveillance methods are indispensable for benchmarking of healthcare-associated infection rates. The National Healthcare Safety Network (NHSN) recently introduced surveillance of ventilator-associated events (VAE), including ventilator-associated conditions (VAC) [1]. This new algorithm is amenable to automated implementation and strives for more consistent interpretation. We assess the feasibility and reliability of automated implementation.

## Materials and methods

Retrospective analysis of an ICU cohort with prospective assessment of ventilator-associated pneumonia (VAP) in two academic medical centers (January 2011 to June 2012). The algorithm was electronically implemented as specified by the NHSN using minute-to-minute ventilator data. Two minor modifications were developed to improve stability and comparability with manual surveillance (10th percentile and intermittent ventilation). Concordance was assessed between the algorithms and prospective surveillance. Attributable mortality of VAC was estimated by multivariable competing-risk survival analysis.

## Results

Two thousand and eighty patients contributed 2,296 episodes of mechanical ventilation (MV). VAC incidence was 10.0/1,000 MV days. Prospective surveillance identified 8 VAP cases/1,000 MV days. The original VAC algorithm detected 32% (38/115) of patients affected by VAP; positive predictive value was 25% (38/152). Using the 10th percentile identified the same number of VAC cases, but only 116 were identical. VAC incidence was 24.9/1,000 MV days with the intermittent ventilation modification. Concordance between the original algorithm and the modified versions was suboptimal. Estimates of attributable mortality varied by implementation: original VAC subdistribution hazard ratio (sdHR) = 4.33, 10th percentile sdHR = 6.26 and intermittent ventilation sdHR = 2.40.

## Conclusions

Concordance between manual VAP surveillance and the VAE algorithm was poor. Although electronic implementation of the VAE algorithm was feasible, small variations considerably altered the events detected and their effect on mortality. Using the current specifications, comparability across institutions using different electronic or manual implementations remains questionable.
